# Modulation of Recognition Memory for Emotional Images by Vertical Vection

**DOI:** 10.3389/fpsyg.2016.00039

**Published:** 2016-02-02

**Authors:** Aleksander Väljamäe, Takeharu Seno

**Affiliations:** ^1^Interaction Design Lab, Human Computer Interaction Group, School of Digital Technologies, Tallinn UniversityTallinn, Estonia; ^2^Faculty of Design, Kyushu UniversityFukuoka, Japan; ^3^Institute for Advanced Study, Kyushu UniversityFukuoka, Japan

**Keywords:** vertical vection, self-motion, recognition memory, emotional valence, arousal level, embodied cognition

## Abstract

Our previous research showed that vertical vection could modulate human mood. We further examined this possibility by using memory recognition task of positive, negative and neutral emotional images with high and low arousal levels. Those images were remembered accidentally while the participants did visual dummy task, and later presented together with novel images during vertical vection-inducing or neutral visual stimuli. The results showed that downward vection facilitated the recognition of negative images and inhibited the recognition of positive ones. These modulations of incidental memory task provide an additional evidence for vection influence on cognitive and emotional processing, and also provide a new paradigm that can be used in future vection and embodied cognition research.

## Introduction

Embodied cognition research has shown that certain body postures and movements can affect cognition and memory (Lakoff and Johnson, 1980; Riskind and Gotay, 1982; Riskind, 1983; Klatzky et al., 1989; Stepper and Strack, 1993; Goldstone and Barsalou, 1998; Barsalou, 1999). For example, compared to slumped posture, an upright body position induces positive mood and positive emotional responses (Riskind and Gotay, 1982; Stepper and Strack, 1993).

Casasanto and Dijkstra (2010) reported that upward and downward hand movements bias the recollection of positive and negative memories. We also obtained similar effect recently in the case of illusory self-motion (vection) perception (Seno et al., 2013a). Vection refers to a subjective perceptual event in which a stationary observer experiences a compelling illusory self-motion by an exposure to large optic flows (Fischer and Kornmüller, 1930). Similarly to Casasanto and Dijkstra (2010), we reported that upward vection can induce more positive emotional valence that bias the recollection of positive memories (Seno et al., 2013a).

Several studies demonstrate that vection influences human cognitive and emotional processing, i.e., memories, mood and arousal level. For example, vection modulates number generation (Seno et al., 2011) and forward/backward vection influence future- or past-oriented thoughts in mental time travel (Miles et al., 2010). In similar vein, Ihaya et al. (2014) reported that stronger vection induces faster mental tempo and also results in larger pupil dilatation typically associated with higher arousal levels. We also recently found that speed of perceived vection also increased an observer’s speech speed (Seno et al., 2013b). A number of studies show that other autonomic responses such as electrodermal activity, heart rate or blood pressure can be also modulated by moving environments inducing vection and/or motion sickness in healthy subjects (Stout and Cowings, 1993; Aoki et al., 2000 and references therein).

Considering these previous research we can hypothesize that vection might alter our emotion state and the underlying affective responses. Previous research has shown that positive and negative emotions can induce positive and negative memory recollection, respectively (mood congruency effect, Bower, 1981; Blaney, 1986). Therefore, we hypothesized that if upward vection could induce positive and downward vection could induce negative emotional states, upward/downward vection might also have the impact on the recognition of positive/negative images respectively. Therefore we conducted an experiment in which we provided participants with image recognition task during the continuous induction of vertical (upward/downward) vection.

## Materials and Methods

### Ethics Statement

The experiment was pre-approved by the ethics committee of Kyushu University and written informed consent was obtained from each participant.

### Participants

Thirty participants (eight males and 22 females) took part in this experiment. Their average age was 22.1 years old (*SE* = 4.39). All participants reported normal vision and had no history of vestibular system diseases. None of them was aware of the purpose of the experiment.

### Apparatus

Stimuli were generated and controlled by two computers (SVT131B11N, Sony, PCG-11212N, Sony) where vection stimuli were presented by a front projector (EB-485W, with 1,024 × 768 pixel resolution at a 60 Hz refresh rate) and the emotional images were controlled by SuperLab and were presented on a 21.5-inch monitor (VPCJ117FJ, Sony). The experiment was conducted in a darkened room.

### Stimuli

**FIGURE 1:**
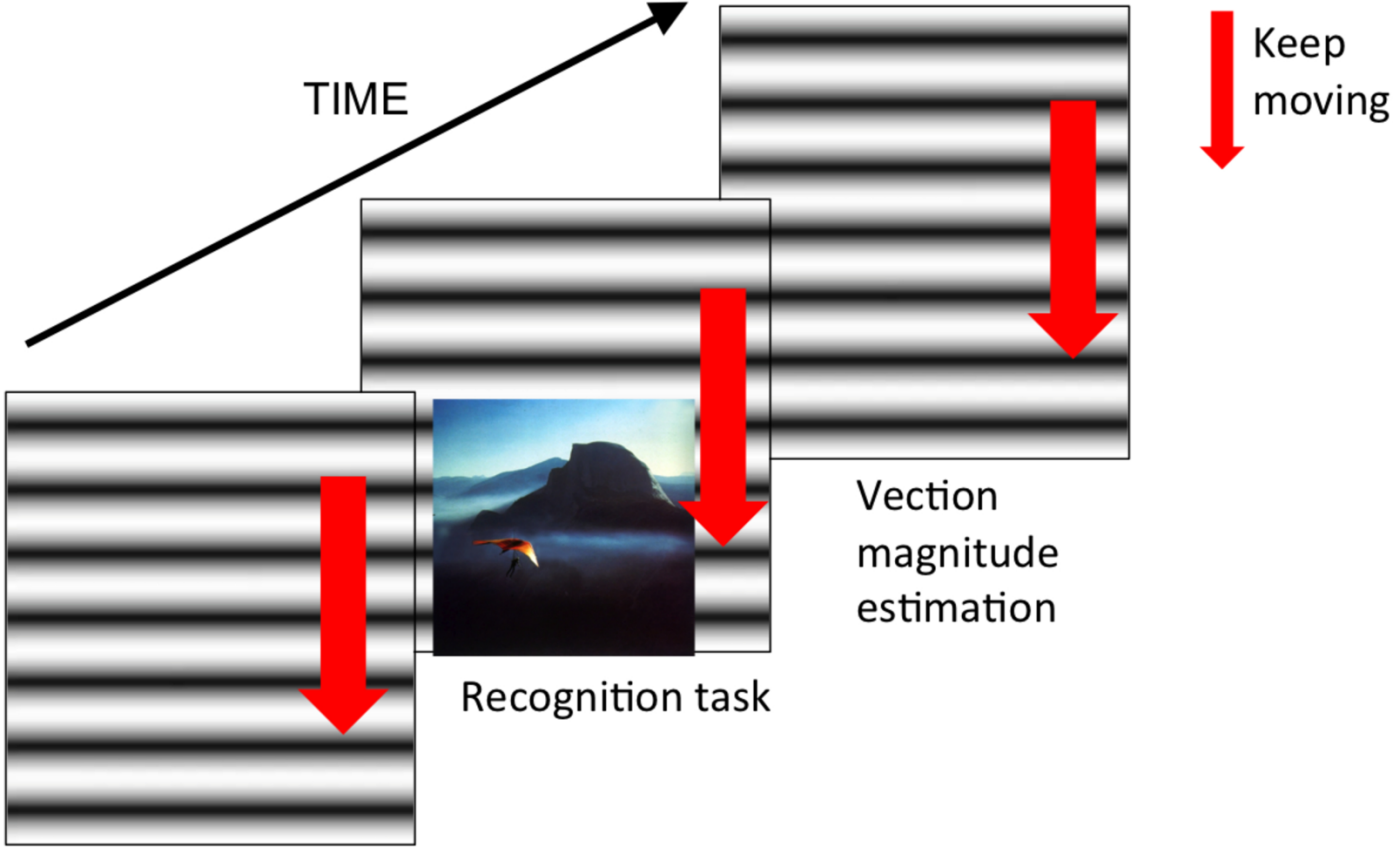
**The schematic illustration of the timing of experimental procedure and stimuli**.

We used a set of images from International Affective Picture System (IAPS) by Lang et al. (2005) and used these for the incidental memory task following the procedures in the previous studies (Bradley et al., 1992; D’Argembeau and Van der Linden, 2004). There were five emotional conditions represented by images with: (1) positive emotional valence with high arousal level, (2) positive emotional valence with low arousal level, (3) negative emotional valence with high arousal level, (4) negative emotional valence with low arousal level and the neutral emotional valence with intermediate arousal level. Each emotional condition had 15 corresponding images. We presented the images at the bottom of the 21.5-inch monitor as shown in **Figure 1**. The image size was 16.4 × 12.3° in visual angle.

In the later recognition task, we added the same number of dummy images corresponding to emotional valence and arousal level to each of the five emotional conditions. Thus we used 150 images in total for the recognition task, 30 per condition.

To create vection stimuli we used upward, downward and static horizontally moving gratings. The stimuli subtended 210° (horizontal) × 122° (vertical) of visual angle at a viewing distance of 57 cm. The spatial frequency of each grating was 0.16 cycle/deg, with the mean luminance of 20.2 cd/m^2^. The Michelson contrast of the gratings was 80%. The velocity of the stimulus was approximately 25°/sec. There was no visual fixation point.

The motion types of vection stimuli were a between-subject factor in our experimental design. The emotional valence and arousal level types were within-subject factors.

### Procedure

As a first part of the experiment, we presented 75 images within a dummy visual task. In this dummy task participants were asked to fixate on the center of the screen. Then one of the 75 images was presented on the screen for 1 s. After the image disappearance some other visual stimuli (some letters) appeared in the center but also at the peripheral parts of the screen. Participants were instructed to respond to those dummy visual stimuli as fast and correct as possible. Although we recorded the performance level for this initial dummy task, these are not relevant for the present study and not reported here. Importantly, 75 IAPS images were presented without the explicit task to be memorized but which triggered participants’ incidental memory.

After this first incidental memory task, we presented vection stimuli to the participants during 10- min period. During this period we explained to the participants about vection sensation, which was defined as a distinct perception of own body motion in relation to the surrounding environment.

Next, as a second part of the experiment, we again presented one of the vection stimuli (upward, downward or static) to each participant group. After the vection stimulus was displayed for 30 s, it was accompanied by the sequential presentation of 150 images (75 target images and 75 dummy images) for the recognition task. Images were presented one by one at the center of the monitor and the participants indicated by a key press whether they already saw these images in the first part of the experiment (the incidental memory task). During this recognition task, vection stimuli were always present at the background (as shown in **Figure 1**). After the recognition task was over, the participants rated the subjective vection strength using a 101-point rating scale ranging from 0 (no vection) to 100 (very strong vection).

## Results

For all analyses alpha level was fixed at 0.05. Greenhouse–Geisser correction was used to correct for unequal variances. All variables were normally distributed according to the Kolmogorov–Smirnov test. Hit Rate (HR) and False-Alarm Rates (FAR) were normalized for each subject so that 100% corresponded to the maximal number pictures recognized for all five types of images. In addition, we also calculated two measures from signal detection theory perspective – namely sensitivity ***d’*** and response bias ***c*** (Stanislaw and Todorov, 1999).

### Magnitude of Vection

**FIGURE 2:**
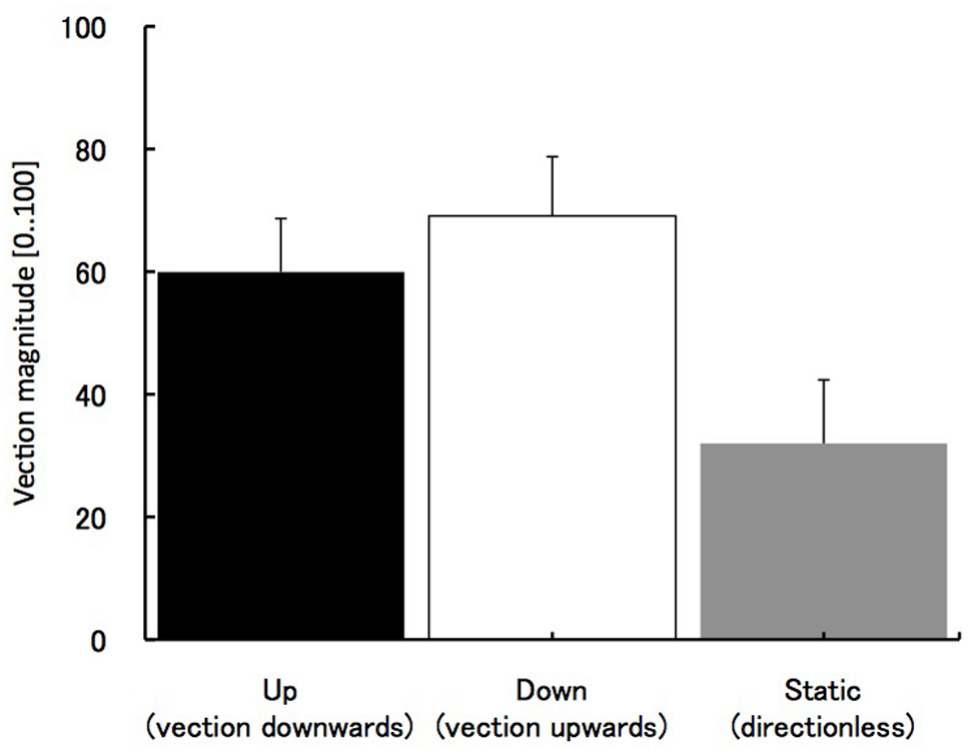
**The results of vection strength.** The horizontal axes indicate three motion types of visual stimulus. Error bars indicate SEs.

Substantial vection was obtained in upward and downward motion stimuli. The average values of vection magnitude ratings in those two conditions were about 60 – *M* = 60 (*SE* = 8.8) for visual motion up (vection downward) and *M* = 69 (*SE* = 9.7) for visual motion down (vection upward). The weaker vection (mean magnitude value of 30 and *SE* = 10.4) was also obtained in static condition (see **Figure 2**). In static condition, no vection was assumed. However, some subjects perceived illusory body motion even in the static condition (see the Discussion for possible explanation of this effect). One-way ANOVA revealed a significant main effect of three stimuli types *F*(2,27) = 4.04, *p* < 0.05, with a significant difference between the static condition and the upward vection (*p* < 0.05, Bonferroni correction for multiple comparisons), but not between the static condition and downward vection (*p* = 0.15, Bonferroni correction).

We also obtained the perceived directions of vection to control if self-motion inducing stimuli were effective. All participants perceived upward or downward vection when presented with visual grating moving down or up respectively. Interestingly, even in the static grating condition, significantly weaker but still reported vection sensation was obtained which was not originally expected. In the post-experimental verbal reports the participants said that some kind of floating perception emerged after observing the static grating for a long time during the recognition task. Our previous research showed that users can experience directionless vection (Seno et al., 2012) or less convincing motion direction when generic motion cues like vibrations are present (Väljamäe et al., 2006).

### Image Recognition Performance and Vection Influence

**FIGURE 3:**
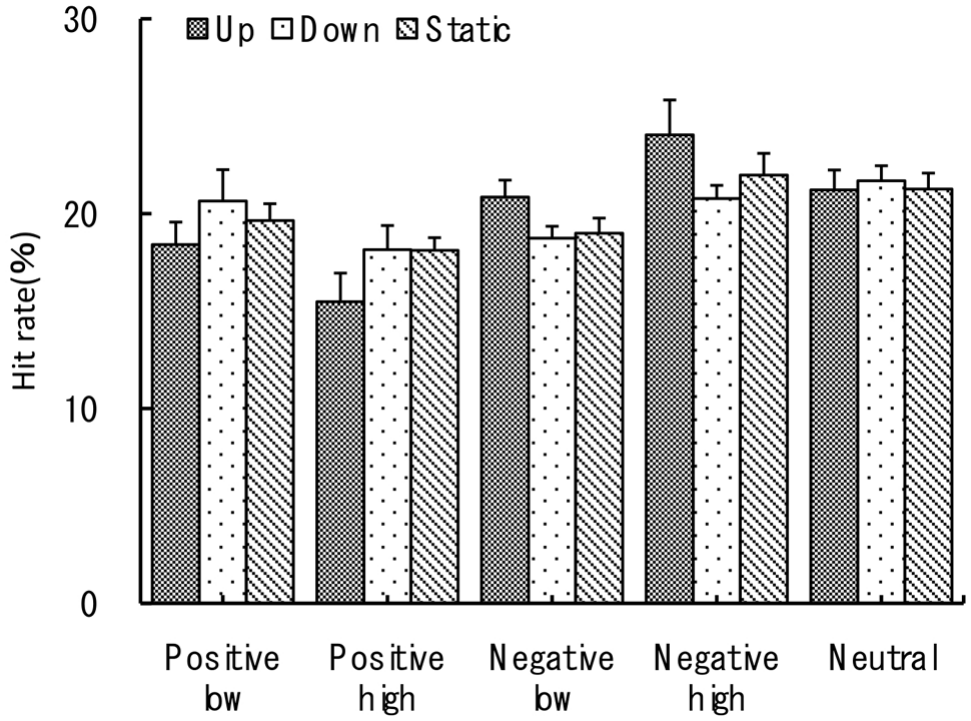
**Normalized hit rate (%).** The recognition task performance for five types of emotional images during viewing vection-inducing visual stimuli moving up, down or being static. Error bars show SE values.

**FIGURE 4:**
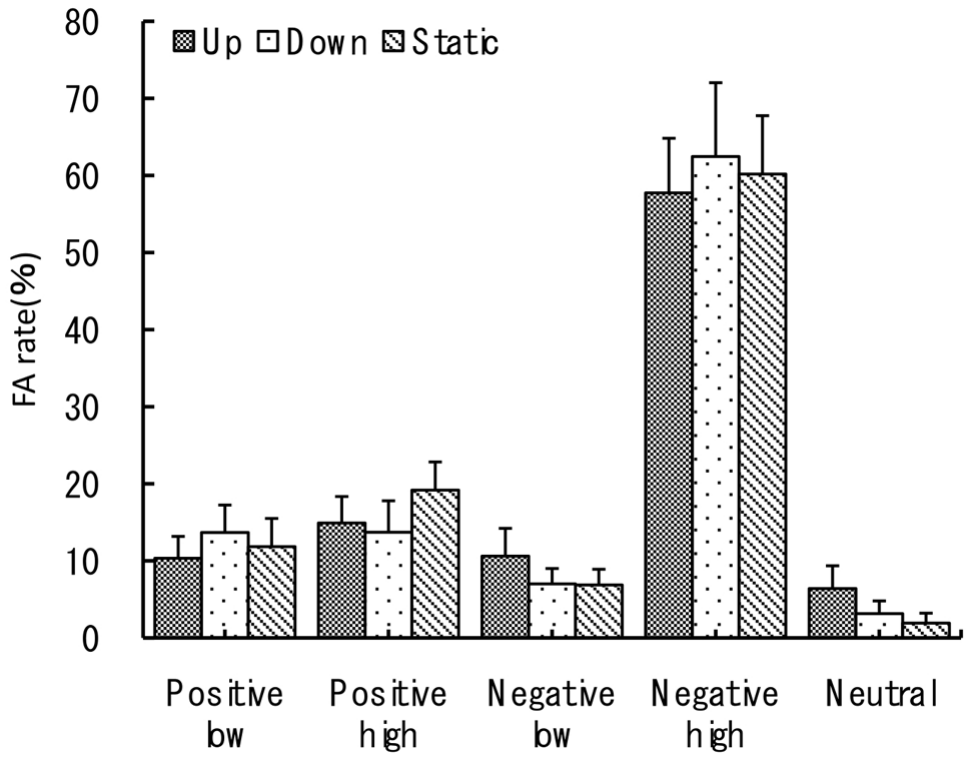
**False-alarm rate.** The recognition task performance for five types of emotional images during viewing vection-inducing visual stimuli moving up, down or being static. Error bars show SE values.

**FIGURE 5:**
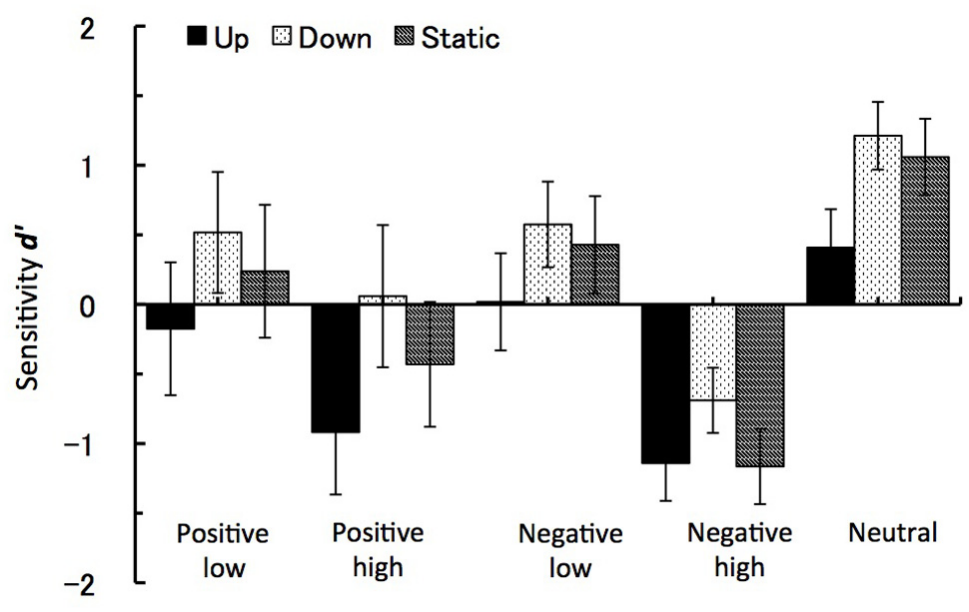
**Sensitivity *d’*.** The recognition task performance for five types of emotional images during viewing vection-inducing visual stimuli moving up, down or being static. Error bars show SE values.

**FIGURE 6:**
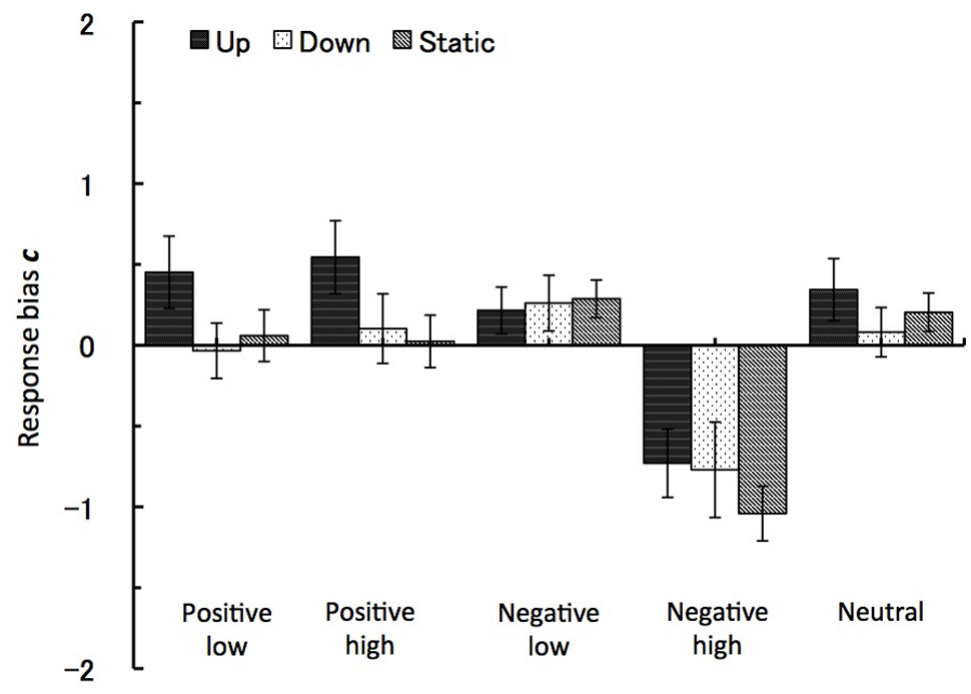
**Response bias *c*.** The recognition task performance for five types of emotional images during viewing vection-inducing visual stimuli moving up, down or being static. Error bars show SE values.

We analyzed separately four measures of recognition performance – hit-rate, false-alarm rate, sensitivity *d’* and response bias *c*. For all four measures we used three-way mixed ANOVAs with the two within-subject factors of emotional valence (negative/positive) and arousal (high/low levels), and with the between-subject factor being the motion type of visual stimuli. The neutral image conditions were excluded from these analyses to allow the exploration of three-way interaction. The neutral image condition values for all four measures are provided as a reference and can be seen in **Figures 3–6**.

For the hit rate three-way ANOVA revealed a significant main effect of emotional valence at *F*(1,27) = 11.02, *p* < 0.005, ${\hat{\eta }}_{\text{p}}^{\text{2}}$ = 0.74. Percentage of recognized negative pictures was slightly but significantly higher than for the positive ones (*M* = 20.9 vs. *M* = 18.4, *p* < 0.05). There was no significant main effect of the arousal level, *F*(1,27) = 0, *p* > 0.78, ${\hat{\eta }}_{\text{p}}^{\text{2}}$ = 0. Two between-factor interactions were also significant. First, the interaction between emotional valence and arousal level was significant at *F*(1,27) = 14.22, *p* < 0.001, ${\hat{\eta }}_{\text{p}}^{\text{2}}$ = 0.35 with maximum recognition rates for high arousal negative pictures (*M* = 22.3, *SE* = 0.7) and minimum rates for high arousal positive ones (*M* = 17.2, *SE* = 0.7), see also **Figure 3** for further details. Second, vection direction (group) and emotional valence interaction was significant at *F*(2,27) = 4.26, *p* < 0.05, ${\hat{\eta }}_{\text{p}}^{\text{2}}$ = 0.24 with stimuli moving up (downward vection) facilitating recognition of negative images and inhibiting positive ones. This was one of the most important findings in this current study together with response bias *c* results provided below. The other interactions were not significant (*p* > 0.05).

For the false-alarm rate three-way ANOVA revealed no significant main effect of three vection types, a significant main effect of emotional valence type at *F*(1,27) = 36.12, *p* < 0.001, ${\hat{\eta }}_{\text{p}}^{\text{2}}$ = 0.57, and a significant main effect of the arousal level at *F*(1,27) = 84.87, *p* < 0.001, ${\hat{\eta }}_{\text{p}}^{\text{2}}$ = 0.76. False recognition percentage was more frequent for negative (*M* = 34.1, *SE* = 1.8) than for positive images (*M* = 13.9, *SE* = 1.6). As for the arousal level effect, high arousal images have lead to a significantly higher FAR percentage (*M* = 38, *SE* = 1.6) than low arousal images (*M* = 14, *SE* = 1.5). Consequently, the interaction between the two factors of emotional valence and arousal level was also significant at *F*(1,27) = 47.76, *p* < 0.001. Here high arousal negative images have lead to the highest FARs and high arousal positive to the lowest FARs as can be seen in **Figure 4**. The other interactions were not significant (*p* > 0.05).

For the sensitivity measure *d’* only the arousal level factor showed a significant difference at *F*(1,27) = 44.33, *p* < 0.001, ${\hat{\eta }}_{\text{p}}^{\text{2}}$ = 0.62. While recognition performance for low arousal images had positive *d’* value (*M* = 0.3, *SE* = 0.2), sensitivity to high arousal images showed possible response confusion reflected by negative *d’* values (*M* = -0.7, *SE* = 0.2). In line with this main effect, the interaction between the arousal level and the emotional valence factors also reached significance at *F*(2,27) = 6.63, *p* < 0.02, ${\hat{\eta }}_{\text{p}}^{\text{2}}$ = 0.2. Here again negative images with high level of arousal lead to the most negative *d’* values (*M* = -1, *SE* = 0.2), as compared to high arousal positive images (*M* = -0.4, *SE* = 0.3) and low arousal images, either positive (*M* = 0.2, *SE* = 0.3) or negative (*M* = 0.3, *SE* = 0.2). **Figure 5** shows the values for all five image groups and for all three motion types.

The analysis of response bias measure ***c*** showed the main effects of the emotional valence and the arousal level factors. For the valence factor the effect was at F(1,27) = 57.06, p < 0.001, ${\hat{\eta }}_{\text{p}}^{\text{2}}$ = 0.68, with the ‘yes’ response bias toward negative images recognition (*M* = -0.3, *SE* = 0.1) as compared to positive images (*M* = 0.2, *SE* = 0.1). The effect from the level of arousal was at *F*(1,27) = 41.47, *p* < 0.001, ${\hat{\eta }}_{\text{p}}^{\text{2}}$ = 0.61, with high arousal images leading to ‘yes’ response bias (*M* = -0.3, *SE* = 0.1) as compared to (*M* = 0.2, *SE* = 0.1). Further demonstrating these effect, the interaction between the valence and arousal factors also reached significance, *F*(1,27) = 41.47, *p* < 0.001, ${\hat{\eta }}_{\text{p}}^{\text{2}}$ = 0.61, with high arousal negative images leading to the strongest recognition response bias (*M* = -0.85, *SE* = 0.1). Importantly for this study, the interaction between visual motion type and emotional valence also reached significance, similarly to the hit rate, with *F*(2,27) = 4.62, *p* < 0.02. As can be seen in **Figure 6**, the group experiencing downward vection (visual grating moving up) significantly increased in their ‘NO’ response bias for positive pictures as compared to same picture type in upward vection or static motion conditions. In other words, downward vection suppressed the recognition of the positive images.

## Discussion

Our study addressed the influence of vertical vection (illusory self-motion) on recognition task of emotional images using three participants groups exposed to visual gratings moving up, down and horizontally (static condition). The substantial vection was obtained in all three visual stimulation conditions. The upward vection induced by downward motion stimuli was the strongest.

Our results showed the significant interaction between vection direction and emotional valence of the images. Stimuli that moved up (downward vection) facilitated recognition of the negative images and inhibited the positive ones in terms of the hit rate. This result well corresponds to our initial hypothesis. Previous studies showed that the downward vection could facilitate negative emotional memories (Seno et al., 2013a). In turn, the negative emotional state then could facilitate the memory of negative images and inhibit the memory for positive images. While we did not do pre- and post-experimental assessment of self-reported emotional state, our results could be explained from the perspective of a well-known “mood congruency effect” (Bower, 1981; Blaney, 1986).

Apart from the hit and false-alarm rate, we also calculated sensitivity ***d’*** and response bias ***c*** measures according to signal detection theory. Similarly to previous studies (Dougal and Rotello, 2007 and references therein), there was a significant response bias toward false recognitions for emotional images, especially for high arousal negative IAPS images. This response was similar for all three types of visual motion. Importantly, there was a significant interaction between the group exposed to motion up (downward vection) and positive pictures. Here the downward vection suppressed recognition of positive pictures with response bias toward ‘NO’ responses.

It should be noted that in our previous studies the effect of upward vection on emotional valence was more prominent than downward vection (Seno et al., 2013a). However, in that study the task was not about recognition of particular images but about the recall of autobiographical episodic memories. Experiment 1 in that study also showed a clear “positivity bias” with nearly double positive events recalled as compared to negative or neutral events. The current methodology allowed avoiding such emotional memory bias.

The found preference for downward vertical vection is not unusual but, unfortunately, not well studied. Linear vection studies often show forward vection bias (Reinhardt-Rutland, 1982; Väljamäe et al., 2008). On the contrary, mixed results have been reported regarding vertical vection bias. In a number of experiments studying perception of different lamellar flows Telford and Frost (1993) showed directional biases for vertical vection in both upward and downward motion conditions. Significant individual differences were also found with over 30% preferring downward and about 16% upward vection. They also noted that individual sensitivity to linear vection is higher than for vertical one, which is less common experience (Telford and Frost, 1993 and references therein). A later study by Giannopulu and Lepecq (1998) did not find significant biases for vertical vection, while Kitazaki and Sato (2003) found bias for vertical vection upward in some specific conditions. It seems that vertical vection is highly susceptible to task conditions and possible biases can be also dependent on individual differences and prior experience of the participants. We believe that the found bias just reflected specificity of the setup. Importantly, this bias does not affect our findings since downward vection affected both positive and negative images recognition in terms of the hit rate.

The dependence of recognition performance on images emotional valence in our study corresponds well to the previous findings. First, the recognition hit rate was highest for the images with negative emotional valence and high arousal level. Better recognition for the negative images has been reported in a number of studies (e.g., Clifford and Scott, 1978; Clifford and Hollin, 1981; Loftus and Burns, 1982). Second, the false-alarm rate was the largest for the images with negative emotional valence and high arousal level. This result also well corresponds to the previous studies that report higher false-alarm rate in the recognition of the negative images (Brainerd et al., 2008, 2010; Dehon et al., 2010; Van Damme and Smets, 2014). For example, Porter et al. (2003) reported that the false-alarm rate would be twice for negative images as compared to positive and neutral images, which corresponds well to our current results. Several previous studies have attributed this effect of high arousal negative images to their biological salience since these might indicate a threat for our survival (e.g., Hansen and Hansen, 1988; Öhman et al., 2001; Keil and Ihssen, 2004; Schimmack and Derryberry, 2005).

Several studies show that high arousal images can be recognized more easily (Cahill and McGaugh, 1995; Cahill et al., 1995; Buchanan et al., 2006). This effect of high arousal level was partially obtained in our current study, but only for negative images. Our results showed a significant interaction between arousal level and emotional valence. Specifically, the high arousal negative images were the most efficiently memorized while the high arousal positive images were the least efficiently memorized ones. It has been noted that the performance of the recognition of negative and positive images is highly affected by the types of task (Christianson, 1992). It seems that high arousal negative images were the most efficient stimuli in our recognition task helping to reveal memory-vection interaction. While we did not access the overall emotional state of the subjects after the experiment, the task could be seen as difficult and rather negative than positive, thus also biasing the overall recognition performance and preference for negative images. Finally, the presentation of the images was below users’ line of sight, which also might contributed to downward vection effect.

Unfortunately, in this experiment we did not explicitly instruct participants to focus on vection inducing stimuli and we did not specifically assess vection chronometry so it is difficult to know to which extent vection was consistent. It is possible that vection perception was not continuous and some negative pictures could be seen by the same person when experiencing vection while others not. In our future experiment we will control when vection can be experienced with clear with- and without vection periods, so we will be able to study the image recognition performance with or without vection.

To summarize, previous vection studies have repeatedly reported that vection can modify human cognition. For example, vection had effects in number generation (Seno et al., 2011), time perception (Seno et al., 2011), day-dreaming (Miles et al., 2010), mood and memory recollection (Seno et al., 2013a), arousal level (Ihaya et al., 2014) and speed of utterance (Seno et al., 2013b). The results of the current study add one more important aspect to this research, namely, the effect of vection on the incidental memory task. This current study also provides a new paradigm that can be used in vection and embodied cognition research. Besides basic research, such new paradigm could be useful in the clinical neuroscience domain where links between self-motion and mood and nervous system disorders are studied (e.g., Crevits and Bosman, 2005).

## Author Contributions

TS designed and executed experiment. AV and TS analyzed data and wrote the article.

## Conflict of Interest Statement

The authors declare that the research was conducted in the absence of any commercial or financial relationships that could be construed as a potential conflict of interest.
